# Immune environment of the brain in schizophrenia and during the psychotic episode: A human post-mortem study

**DOI:** 10.1016/j.bbi.2021.07.017

**Published:** 2021-10

**Authors:** Livia J. De Picker, Gerardo Mendez Victoriano, Rhys Richards, Alexander J. Gorvett, Simeon Lyons, George R. Buckland, Tommaso Tofani, Jeanette L. Norman, David S. Chatelet, James A.R. Nicoll, Delphine Boche

**Affiliations:** a Collaborative Antwerp Psychiatric Research Institute, University of Antwerp, Antwerp, Belgium; b University Psychiatric Department Campus Duffel, Duffel, Belgium; c Clinical Neurosciences, Clinical and Experimental Sciences, Faculty of Medicine, University of Southampton, Southampton, UK; d Psychiatry Unit, Health Science Department, University of Florence, Florence, Italy; e Histochemistry Research Unit, Clinical and Experimental Sciences, Faculty of Medicine University of Southampton, Southampton, UK; f Biomedical Imaging Unit, Southampton General Hospital, University of Southampton, Southampton, UK; g Department of Cellular Pathology, University Hospital Southampton NHS Foundation Trust, Southampton, UK

**Keywords:** Schizophrenia, Psychosis, Microglia, Perivascular macrophages, T lymphocytes, Human brain

## Abstract

A causal relationship between immune dysregulation and schizophrenia has been supported by genome-wide association studies and epidemiological evidence. It remains unclear to what extent the brain immune environment is implicated in this hypothesis. We investigated the immunophenotype of microglia and the presence of perivascular macrophages and T lymphocytes in *post-mortem* brain tissue. Dorsal prefrontal cortex of 40 controls (22F:18M) and 37 (10F:27M) schizophrenia cases, of whom 16 had active psychotic symptoms at the time of death, was immunostained for seven markers of microglia (CD16, CD32a, CD64, CD68, HLA-DR, Iba1 and P2RY12), two markers for perivascular macrophages (CD163 and CD206) and T-lymphocytes (CD3). Automated quantification was blinded to the case designation and performed separately on the grey and white matter. 3D reconstruction of Iba1-positive microglia was performed in selected cases. An increased cortical expression of microglial Fcγ receptors (CD64 *F* = 7.92, *p* = 0.007; CD64/HLA-DR ratio *F* = 5.02, *p* = 0.029) highlights the importance of communication between the central and peripheral immune systems in schizophrenia. Patients in whom psychotic symptoms were present at death demonstrated an age-dependent increase of Iba1 and increased CD64/HLA-DR ratios relative to patients without psychotic symptoms. Microglia in schizophrenia demonstrated a primed/reactive morphology. A potential role for T-lymphocytes was observed, but we did not confirm the presence of recruited macrophages in the brains of schizophrenia patients. Taking in account the limitations of a *post-mortem* study, our findings support the hypothesis of an alteration of the brain immune environment in schizophrenia, with symptomatic state- and age-dependent effects.

## Highlights

• Low-grade neuroinflammation is state and age-dependent in chronic schizophrenia.
• 3D microglial morphology shows primed/reactive microglia in chronic schizophrenia.
• Cerebral T lymphocytes recruitment consistent with a role for adaptive immunity in chronic schizophrenia.

## 1 Introduction

Schizophrenia is a severe chronic mental disorder affecting approximately 1% of the world population, hallmarked by episodes of psychotic symptoms (i.e. hallucinations, delusions and disorganized thought or behaviour) emerging in adolescence or early adulthood. The illness is syndromic and heterogeneous and while there are well-established criteria in place for the diagnosis of schizophrenia, its causes are still unclear. This is partly due to the disease complexity that cannot be replicated in animal models (McCullumsmith et al., 2014) and only explored in the human brain, and its highly variable clinical course, presenting both a progressive and relapsing-remitting condition with fluctuating levels of psychotic symptoms in the majority of patients.

Genome-wide association studies (GWAS), epidemiological studies and longitudinal observational studies have identified immune system disturbances at the core of both neurodevelopmental and neurodegenerative processes in schizophrenia (Bayer et al., 1999, Horvath and Mirnics, 2014, Monji et al., 2009, Monji et al., 2013, Purcell et al., 2009, Smigielski et al., 2020). Increased risk for schizophrenia is conferred by interplay of early environmental exposure to proinflammatory immune responses and risk alleles in immune genes (Brown et al., 2004, Corvin and Morris, 2014, Ellman et al., 2010, Purcell et al., 2009), the strongest of which is in the major histocompatibility complex (MHC) locus on chromosome 6 (Purcell et al., 2009), encoding human leukocyte antigen (HLA) (Schizophrenia Working Group of the Psychiatric Genomics, 2014). In the central nervous system, HLA is expressed by microglia, the immune cells of the brain (De Picker et al., 2017). These findings have been interpreted to characterize hyperactive complement-mediated microglial synaptic pruning as a key mechanism of schizophrenia pathophysiology. (Sekar et al., 2016). Hence, abnormal activation of microglia is central to the hypothesis that dysregulated immune responses during adult life cause symptom development and structural and functional brain abnormalities through disruptions of synaptic plasticity in schizophrenia (De Picker et al., 2017). Transcriptomic changes in an inflammatory response pathway were found in brain tissue of around 40% of schizophrenia patients (Fillman et al., 2013), while some microglial transcriptomic expression patterns have been found to be downregulated in schizophrenia brain tissue (De Picker et al., 2017, Gandal et al., 2018). Empirical evidence of abnormal microglial activity in schizophrenia derives from *post-mortem* neuropathological studies and *in vivo* positron emission tomography (PET) with a ligand of the 18kDA translocator protein (TSPO) (De Picker et al., 2017). However, both lines of study have generated conflicting and heterogeneous results and have been unable to sufficiently differentiate between trait markers underpinning the schizophrenia endophenotype, versus state-specific changes associated with a symptomatic psychotic episode. Study of peripheral immune biomarkers indicates a distinct immunological state during a psychotic episode: patients experiencing an acute relapse or first episode of psychosis manifest innate immune system activation (increased neutrophil and monocyte counts), increased CD4/CD8 lymphocyte ratios and increased levels of pro-inflammatory cytokines (IL1β, IL6, IL8, TNFα and TGFβ) compared to patients in remission (De Picker et al., 2019a, De Picker et al., 2017, Miller et al., 2011, Steiner et al., 2020, Yuan et al., 2019). Importantly, state-specific immune changes could affect treatment outcomes (Nitta et al., 2013): *meta*-analyses of RCTs have pointed out that while inpatients and first-episode patients may benefit from treatment with non-steroidal anti-inflammatory drugs, outpatients and chronic patients probably do not.

It remains unclear to what extent the brain immune environment is implicated in immune dysregulation in schizophrenia. While *in vivo* PET neuroimaging has the advantage of allowing longitudinal study of the dynamics of glial activation, inconsistent results among cross-sectional PET studies have raised questions about the validity of the TSPO tracer as biomarker and the potential role of immunosenescence and symptomatic state on microglial activity in schizophrenia (Boche et al., 2019, Conen et al., 2020, De Picker et al., 2019b, Plavén-Sigray et al., 2020, Sneeboer et al., 2020). Moreover, comparability between studies is limited due to methodological differences such as the brain areas explored, use of different microglial markers and TSPO tracers (De Picker and Morrens, 2020, De Picker et al., 2017). High-quality neuropathological research is urgently needed to study the true nature and role of brain immune cells in schizophrenia (De Picker et al., 2017, Monji et al., 2009).

In this hypothesis-generating human neuropathological study, we explored the immunophenotype of microglia using markers associated with specific functions of microglia/macrophages (Supplementary Table 1), reconstructed 3D microglia for detailed morphological assessment and analysed perivascular macrophage populations and T lymphocytes in schizophrenia. Based on our earlier findings with TSPO PET in a longitudinal clinical study of patients during an acute psychotic episode (De Picker et al., 2019b), we also tested the hypothesis of age- and symptomatic state-dependent effects on microglial changes.

## 2 Methods

### 2.1 Cases

Thirty-seven cases with a confirmed diagnosis of schizophrenia or schizoaffective disorder (Sz) and 40 controls (Ctrl) were obtained from the Corsellis Collection (Table 1). In the Sz group, evidence of a current psychotic episode (i.e. delusions, hallucinations, grossly disorganised speech or behaviour within one month before the time of death documented in clinical case records) was present in 16 (Sz+) and absent in 21 (Sz-) subjects. Dorsal prefrontal cortex was selected for investigation, as an area showing neuroimaging abnormalities with reduction of the grey matter volume in chronic schizophrenia (Kikinis et al., 2010). Cases with any other significant brain pathologies such as stroke, tumour or traumatic brain injury were excluded from the study. Controls with no history of neurological or psychiatric disease, or cognitive impairment were matched for age, gender and *post-mortem* delay as closely as possible.

**Table 1:** Demographic, clinical and *post-mortem* characteristics of control and schizophrenia cases.

Cases	Ctrl (n = 40)	Sz (n = 37)	Test statistic	P value
Gender	22F:18M	10F:27M	X^2^ 0.013	0.020
Age at death (years, mean ± SD (age range))	76.28 ± 22.13 (23–100)	62.05 ± 17.82 (21–94)	t −3.10	0.003
Post-mortem delay (hours, mean ± SD)	47.35 ± 32.75	42.50 ± 22.69	t −0.63	0.530
Evidence of psychotic symptoms at time of death		Present 16/37 (43.2%) Absent 21/37 (56.8%)		
Age of onset (years, mean ± SD)		32.03 ± 10.89		
Duration of illness (years, mean ± SD)		30.36 ± 18.31		
Cause of death				
*Cardiovascular disease*	31/40 (77.5%)	13/37 (35.1%)		
*Infection/inflammation*	6/40 (15.0%)	14/37 (37.8%)		
Trauma*	3/40 (7.5%)	8/37 (21.7%)		
Others*	0/40 (0.0%)	2/37 (5.4%)		

*Ctrl*, neurologically/cognitively normal controls; *Sz*, Schizophrenia cases; *F*, female, *M*, male; *SD*, standard deviation. *Trauma in Ctrl: multiples injuries (unspecified), injury intra-abdominal organs, foreign body in respiratory tract. *Trauma in Sz: 6 suicides, hypothermia, foreign body in respiratory tract. *Other causes of death: liver disease, unknown.

### 2.2 Ethics

Ethical approval was provided by BRAIN UK, a virtual brain bank which encompasses the archives of neuropathology departments in the UK and the Corsellis Collection, ethics reference 14/SC/0098.

### 2.3 Immunohistochemistry

Sections were prepared from paraffin embedded blocks processed at the time of the original *post-mortem* macroscopic brain examination, after fixation of the whole brain in formalin for, typically 6 weeks. For the assessment of protein expression, 6 μm thick sections were dewaxed and rehydrated before treatment with hydrogen peroxide and methanol solution to block endogenous peroxidases. Appropriate antigen retrieval steps were performed before application of the primary antibodies (Supplementary Table 1). Biotinylated secondary antibodies (Dako, Denmark) were visualized using avidin–biotin–peroxidase complex method (Vectastain Elite) with 3,3′-diaminobenzidine as chromogen (Vector Laboratories, UK). Sections were counterstained with haematoxylin, dehydrated and mounted in DePeX (VWR International, UK). Slides were immunolabelled in batches, with each batch containing cases from both groups together with appropriate negative controls and tonsil as positive control tissue as previously published (Rakic et al., 2018, Zotova et al., 2013).

For 3D analysis, 50 μm thick mounted sections were immunostained with Iba1 antibody (Wako, Japan) followed by incubation with secondary antibody anti-rabbit Alexa Fluor 635 (Thermofisher Scientific) to allow identification of microglia and their processes by fluorescent microscopy. The slides were counterstained with 4′,6-diamidino-2-phenylindole (DAPI, 1:75 dilution) to visualise nuclei to identify microglial cell bodies, and mounted in Mowiol (Sigma Aldrich, UK). The stained slides were examined under a confocal microscope (Leica TCS SP8). Using Leica LASX software, five Z-stacks (each one composed of 100 focal planes) were taken from the grey matter of each of the sections under a x63 objective.

### 2.4 Quantification

Quantification was blinded to case designation. Slides were scanned using the Olympus VS110 Virtual Slide Microscopy system under a 20x objective (Olympus America Inc). For each case, 30 regions of interest of 0.25 mm^2^ each, were extracted separately in grey and white matter using Olympus VS-Desktop software. Quantitative image analysis was carried out using ImageJ (Wayne Rasband, NIH, USA). For each antibody, a specific threshold was determined to quantify the area fraction of each image labelled by the antibody and expressed as protein load (%), and the mean value was calculated for each case and each antibody. HLA-DR is one of the genetic risk factors identified for schizophrenia (Schizophrenia Working Group of the Psychiatric Genomics, 2014) and ratios with HLA-DR in the denominator were calculated to assess the proportion of microglial cells with a specific phenotypical feature associated with this immune risk factor.

CD163 + and CD206 + perivascular macrophages and CD3 + T lymphocytes were analysed as present or absent based on assessment of the whole section under a × 10 objective. Macrophages were assessed as recruited within the parenchyma and T lymphocytes in relation to the perivascular spaces, parenchyma of the grey and white matter and meninges, with data presented as the percentage of cases with T lymphocytes present in each group.

### 2.5 3D microglial reconstruction and assessment of morphological features

In order to be representative of their groups, cases were selected based on their Iba1 load being in close range to the mean. Per case, ten microglia were selected in grey matter from four control and five schizophrenia cases and reconstructed in 3D using Imaris (Bitplane) software (Oxford Instruments, UK). Cells were selected on the basis that they were centrally located in the XY perspective of the stack obtained by confocal microscope, with a complete Iba1 + cell body and processes encompassing a DAPI-stained nucleus. The cell body was constructed using the function “Surfaces” with the contour manually drawn for each focal plane before conversion to a 3D-object using the function “Create surface”. The microglial processes were semi-automatically traced using the functions “AutoPath” (computes the path of the signal given defined starting and ending points) and “AutoDepth” (automatic computation of the depth).

Features measured of the microglial morphology included: (1) cell body volume (µm^3^); (2) cell body sphericity (ratio of cell body surface area to the surface area of a sphere of the same volume); (3) number of primary processes defined as the number of processes arising from the cell body; (4) length of primary processes (µm), excluding all branches; (5) number of branch points, counting any junction in the process network; (6) branch length (µm) calculated as sum of all branches and sub-branches, excluding the primary process (the longest single path from the cell body); (7) and straightness of the primary processes (ratio of the shortest distance from start to end point of the process to the actual length of the process).

### 2.6 Statistical analysis

Baseline differences in demographic parameters between groups were examined by two-tailed independent t-tests for continuous variables and Fisher’s exact test for categorical variables. All data are presented as mean ± standard deviation (SD) unless stated otherwise.

Normality of distribution across each group was assessed by examination of quantile–quantile plots (not shown). Non-normally distributed variables were log transformed. Multiple linear regression analyses were run to assess group (=2 levels: Ctrl and Sz) differences for microglial markers, with age as covariate. A subgroup analysis was performed for schizophrenia cases divided according to the presence of psychotic symptoms at time of death (multiple linear regression analyses with group = 3 levels: Ctrl, Sz- and Sz+), with planned intergroup comparisons between Sz + vs. Ctrl and Sz + vs. Sz- using Dunnett’s test for multiple comparisons. Correlations were performed based on the normality of the data using Pearson's or Spearman’s test. For the CD3 + T lymphocytes, Fisher’s exact test was used for comparisons between groups.

To rule out confounding by group age and sex imbalances, a posthoc sensitivity analysis was carried out with exclusion of 9 control cases to match groups for age at time of death, sex and year of death. All reported conclusions remained robust, except for the finding in leptomeningeal CD3 + T-cells.

Analyses were performed with IBM SPSS Statistics (SPSS Inc. Chicago IL) and JMP version 14.3. *P* < 0.05 for intergroup comparisons and 0.01 for correlations were considered statistically significant (Rakic et al., 2018).

## 3 Results

### 3.1 Study population characteristics

The controls were significantly older than the Sz cases (t −3.1; DF 75; *p* = 0.003). There were more males among the Sz cases (2-Tail Fisher’s exact test *p* = 0.020). There was no significant between-group difference for duration of *post-mortem* interval or year of death). Causes of death differed, with Sz cases demonstrating more traumatic deaths (including suicide) and systemic infection, and fewer cardiovascular events. Five patients suffered from late-onset schizophrenia (illness onset > 45 years old). Four patients died after duration of illness < 5 years. Most patients were medicated at the time of death, but neither current chlorpromazine equivalent dosage nor lifetime cumulative exposure to antipsychotics could be reliably determined for all cases.

### 3.2 Microglial markers

**Fig. 1:**
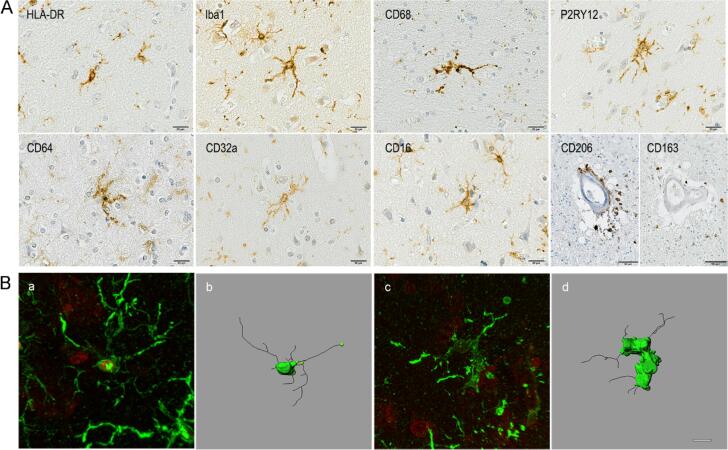
(A) Illustrations of the immunostaining obtained with the different microglial/macrophages markers in schizophrenia. Counterstaining: Haematoxylin. Scale bar = 20 μm, CD206 and CD163 scale bar = 50 μm. (B) Illustration of 3D reconstructed Iba1 + microglial cell in (a-b) control brain and (c-d) schizophrenia brain. Original confocal stack images (a, c) use to reconstruct the cell (b, d). Scale bar = 10 μm.

Multiple markers associated with specific microglial functions were explored (Fig. 1A) including: HLA-DR, associated with antigen-presentation of non-self-recognition (Minett et al., 2016) and identified as a risk factor for schizophrenia (Purcell et al., 2009, Schizophrenia Working Group of the Psychiatric Genomics, 2014); Iba1, a marker of microglial motility (Franco-Bocanegra et al., 2019); P2RY12 a purinergic receptor involved in microglial motility (Franco-Bocanegra et al., 2019) identified as one of the physiological/homeostatic markers of microglia (Butovsky et al., 2014); and CD68, a lysosomal/endosomal-associated transmembrane glycoprotein associated with phagocytosis (Minett et al., 2016). FcγRs, as central effectors of immunoglobulin (Ig)G-mediated immune responses (Nimmerjahn et al., 2015) were examined using CD64 (FcγRI), a high-affinity activating receptor; CD32a (FcγRIIa) and CD16 (FcγRIII), both low-affinity activating receptors; and CD32b (FcγRIIb) a low affinity inhibitory receptor. HLA-DR, Iba1, CD68, P2RY12, CD64, CD16 and CD32a immunolabelled microglia and perivascular macrophages; whereas CD32b was detected only in neurons.

### 3.3 Schizophrenia cases vs. Controls

**Fig. 2:**
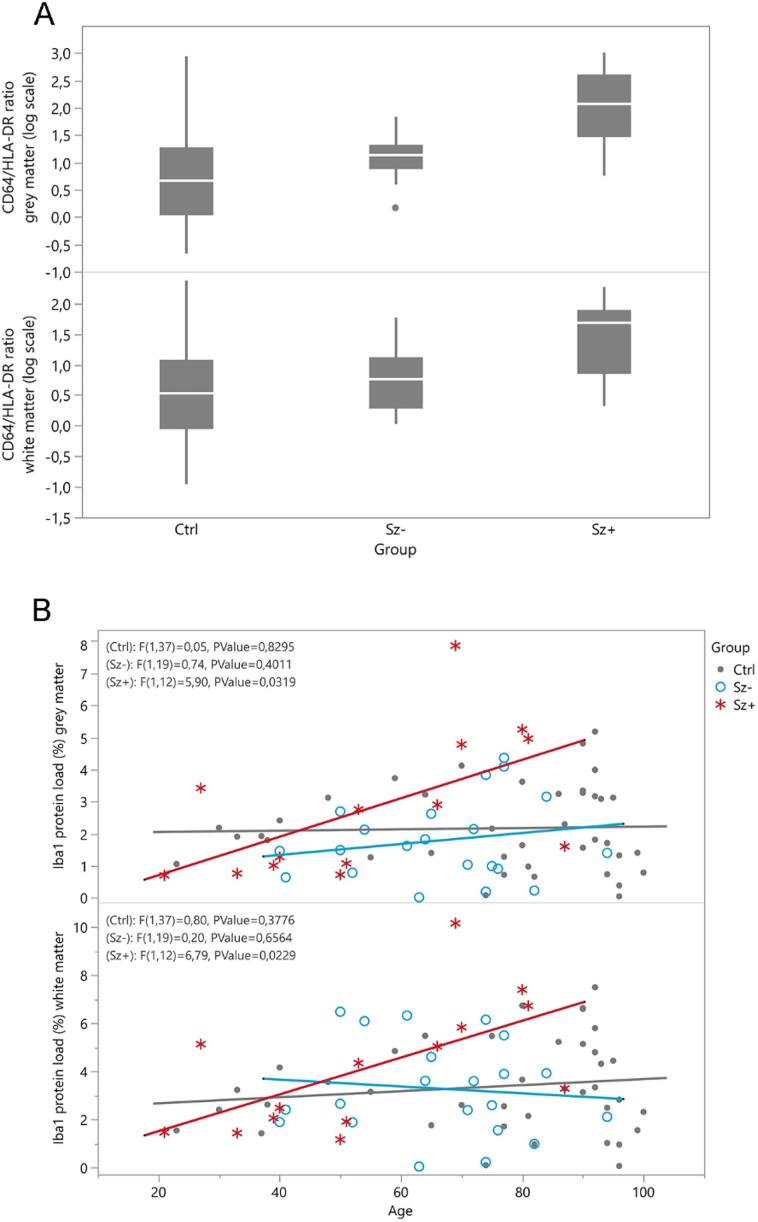
(A) Boxplots of CD64/HLA-DR ratio for each subgroup in grey matter (top panel) and white matter (bottom panel). (B) Linear association between age and Iba1 protein load (%) for each subgroup in grey matter (top panel) and white matter (bottom panel), including F-test for line of fit. Ctrl = control subjects, Sz- = schizophrenia cases without evidence of psychosis at death, Sz+ = schizophrenia cases with evidence of psychosis at death.

Quantification showed a significantly increased CD64 load in grey matter in the schizophrenia group compared to controls (*F* = 7.921 *p* = 0.007; Table 2) and a significantly higher CD64/HLA-DR ratio (Sz median 17.4 (IQR 7.9, 44.2) vs. Ctrl 4.6 (1.09, 18.3) *F* = 5.02, *p* = 0.029**,** Fig. 2A). No age-adjusted differences between schizophrenia cases and controls were observed for the other markers, including HLA-DR (Table 2). However, a significant age effect was observed for HLA-DR (grey matter *F* = 13.51, *p* < 0.0015; and white matter *F* = 8.67, *p* = 0.004), CD16 (grey matter *F* = 10.93, *p* = 0.001; and white matter *F* = 20.59, *p* < 0.001) and CD32b (white matter *F* = 8.84, *p* = 0.004), with higher protein loads in older subjects of both groups.

**Table 2:** Raw data for the inflammatory protein loads (%) in cortical grey matter and white matter of control and schizophrenia cases, and with the schizophrenia cases subcategorised as with or without psychosis at the time of death.

Grey Matter	Ctrl (n = 40)	Sz (n = 37)	P value*	Sz- (n = 21)	Sz+ (n = 16)	P value**
HLA-DR	0.657 ± 1.064 (1)	0.234 ± 0.308 (0)	0.996	0.271 ± 0.264 (0)	0.186 ± 0.362 (0)	0.080
CD68	0.179 ± 0.070 (2)	0.192 ± 0.066 (0)	0.380	0.200 ± 0.073(0)	0.182 ± 0.055 (0)	0.664
Iba1	2.169 ± 1.297 (1)	2.192 ± 1.750 (2)	0.612	1.792 ± 1.274 (0)	2.791 ± 2.207 (2)	0.004
P2RY12	1.760 ± 1.550 (0)	2.143 ± 1.800 (1)	0.697	2.111 ± 1.73 (0)	2.188 ± 1.870 (1)	0.604
CD64	1.893 ± 2.066 (8)	2.764 ± 1.819 (6)	0.007	2.606 ± 1.512 (0)	3.098 ± 2.402 (6)	0.999
CD32a	2.450 ± 2.173 (3)	2.356 ± 1.701 (3)	0.519	2.285 ± 1.170 (0)	2.470 ± 2.378 (3)	0.497
CD16	0.639 ± 0.944 (6)	0.618 ± 0.876 (2)	0.819	0.588 ± 0.715 (0)	0.662 ± 1.103 (2)	0.999
CD32b	1.977 ± 3.549 (5)	1.930 ± 3.284 (5)	0.932	1.578 ± 3.055 (5)	2.282 ± 3.563 (0)	0.720

White matter						
HLA-DR	1.802 ± 2.608 (1)	0.819 ± 0.953 (0)	0.843	0.984 ± 0.884 (0)	0.602 ± 1.026 (0)	0.074
CD68	0.244 ± 0.063 (2)	0.254 ± 0.068 (0)	0.639	0.265 ± 0.061 (0)	0.238 ± 0.076 (0)	0.290
Iba1	3.376 ± 1.914 (1)	3.641 ± 2.304 (2)	0.322	3.283 ± 1.990 (0)	4.178 ± 2.696 (2)	0.022
P2RY12	1.500 ± 1.511 (0)	1.647 ± 1.638 (1)	0.744	1.511 ± 1.591 (0)	1.837 ± 1.739 (1)	0.755
CD64	3.197 ± 2.087 (8)	4.185 ± 3.147 (7)	0.145	4.402 ± 2.760 (0)	3.678 ± 4.055 (7)	0.161
CD32a	1.597 ± 1.456 (4)	1.646 ± 1.258 (4)	0.706	1.465 ± 1.072 (1)	1.924 ± 1.504 (3)	0.611
CD16	1.027 ± 0.956 (6)	0.986 ± 1.298 (3)	0.578	1.084 ± 1.510 (0)	0.840 ± 0.928 (3)	0.693
CD32b	0.127 ± 0.239 (6)	0.157 ± 0.321 (5)	0.181	0.113 ± 0.220 (4)	0.206 ± 0.410 (1)	0.720

Ctrl: neurologically/cognitively normal controls; Sz: schizophrenia cases; Sz+: schizophrenia with psychosis at the time of death, Sz-: schizophrenia cases without psychosis at the time of death. Values are presented as mean ± SD (reported missing cases); *p-value by linear regression analysis, with age as covariate. **Dunnett-adjusted p-value by linear regression analysis, with age as covariate.

### 3.4 Schizophrenia with psychosis vs. Schizophrenia without psychosis

We observed an age-dependent increase in Iba1 load in the schizophrenia cases with psychosis at death which was not observed in schizophrenia without psychosis and controls (interaction between group (3 levels) and age: *F* = 3.66, *p* = 0.031 in grey matter; *F* = 3.03, *p* = 0.055 in white matter; Dunnett’s test for multiple comparisons Sz+ vs. Ctrl GM *t* = 2.86, adjusted *p* = 0.001; WM *t* = 2.69; adjusted *p* = 0.016; Sz+ vs. Sz- grey matter *t* = 3.23, adjusted *p* = 0.004; white matter *t* = 2.56, adjusted *p* = 0.022; Fig. 2B). There was also a significantly increased CD64/HLA-DR ratio in the presence of psychosis compared to controls (grey matter and white matter) and compared to patients without psychosis (grey matter) (Dunnett’s test for multiple comparisons Sz+ vs. Ctrl grey matter *t* = 3.78, adjusted *p* < 0.001; white matter *t* = 2.36, adjusted *p* = 0.037; Sz+ vs. Sz- grey matter *t* = 2.95, adjusted *p* < 0.001; white matter *t* = 1.88, adjusted *p* = 0.103, Fig. 2A). There were no age-adjusted subgroup differences for the other markers (Table 2). Significant age effects were again observed for HLA-DR (grey matter *F* = 11.14, *p* = 0.001; white matter *F* = 6.75, *p* = 0.011), CD16 (grey matter *F* = 10.55, *p* = 0.002; white matter *F* = 19.08, *p* < 0.001) and CD32b (white matter *F* = 9.10, *p* = 0.004) across all subgroups.

### 3.5 Association analysis

To evaluate possible interactions between the different markers, association analysis was carried out within both groups and for grey and white matter (Supplementary Table 2). In summary, in the schizophrenia group, associations of CD68 vs. Iba1, of CD68 vs. CD32b, of Iba1 vs*.* P2RY12 and of HLA-DR vs. CD32b were lost; whereas relations of CD16 with CD32a and CD32b were detected in the grey matter. In the white matter, HLA-DR vs. CD16, HLA-DR vs. CD32b and Iba1 vs. CD32b were lost; whereas association of CD16 with Iba1 and CD32b were observed. All markers correlated between grey and white matter regardless of subgroup, except for CD64 and CD32a for which grey-white matter associations were lost in schizophrenia patients (Supplementary Table 3).

### 3.6 Perivascular macrophages

Potential brain infiltration by perivascular macrophages was explored using CD163 and CD206 immunomarkers specifically associated with perivascular macrophages. We did not observe CD163 + or CD206 + cells outside the perivascular regions in either group (Fig. 1A).

### 3.7 T Lymphocytes

The pan-T cell marker CD3 was used to explore T lymphocyte recruitment. Significantly fewer schizophrenia cases had T lymphocytes in the leptomeninges than controls (Ctrl 28.2% vs. Sz 8.3%; *X^2^*(1, N = 75) = 4.87, *p* = 0.038). A trend toward more schizophrenia cases with lymphocytes in the grey matter parenchyma was detected (Ctrl 46.2% vs. Sz 69.4%; *X^2^*(1, N = 75) = 4.15, *p* = 0.061) (Supplementary Table 4).

### 3.8 Microglial morphology

3D reconstruction and quantitative assessment of morphological features of Iba1 + microglia was performed (Fig. 1B). Schizophrenia cases showed a significantly larger microglial cell body volume (Ctrl 171.00 μm^3^ vs*.* Sz 238.50 μm^3^; *p* = 0.009, Mann-Whitney U Test) and a decrease in cell body sphericity (Ctrl 0.72 vs*.* Sz 0.63; *p* < 0.001, T-test) (Table 3). There were no significant differences for the other features measured.

**Table 3:** Microglial morphology assessment in control and schizophrenia cases.

	Ctrl	Sz	P value
Cell body volume (μm^3^)	197.850 ± 12.787	305.780 ± 30.204	*0.009*
Cell body sphericity	0.719 ± 0.014	0.626 ± 0.017	*<0.001*
Number of primary processes	5.780 ± 0.339	5.660 ± 0.279	0.852
Length of primary processes (μm)	21.776 ± 1.353	23.156 ± 1.399	0.403
Number of branch points	23.280 ± 1.642	22.120 ± 2.617	0.094
Branch length (μm)	162.947 ± 12.835	177.811 ± 22.279	0.495
Straightness of the primary processes	0.810 ± 0.124	0.800 ± 0.120	0.691

Values are presented as mean ± SD.

Significant p value in italic.

Ctrl: neurologically/cognitively normal controls; Sz: schizophrenia cases.

Ctrl group: 40 microglial cells assessed in a total of 4 cases; Sz group: 50 microglial cells assessed in a total of 5 cases.

## 4 Discussion

The novelty of our study resides in the wide range of markers to explore the immune environment of the brain in schizophrenia, with 3D morphological reconstruction of microglia, and the potential roles of perivascular macrophages and T lymphocytes. Previous immunohistochemistry studies of microglia in the brains of schizophrenia patients have been limited to HLA-DR, and to a lesser extent CD68, Iba1, CD11b and MHC I or II, with highly inconsistent results (Trepanier et al., 2016). With a larger sample size than most previous *post-mortem* studies of schizophrenia patients, we had sufficient statistical power to generate new hypotheses on the microglial phenotype in schizophrenia, and to investigate the mediating effects of age and psychotic symptoms at the time of death.

### 4.1 Microglial-related immunomarkers in schizophrenia

Increased expression of CD64 was detected in the dorsal prefrontal cortex in schizophrenia, regardless of the presence of psychotic symptoms or subjects’ age. CD64 is one of the activating FcγRs (FcγRI) with a high affinity for monomeric IgG (Guilliams et al., 2014). Microglia express CD64 (Minett et al., 2016, Rakic et al., 2018), potentially reflecting low IgG concentrations within the brain parenchyma under healthy conditions (Diamond et al., 2013). Only approximately 0.1% of circulating antibodies enter the brain via passive diffusion (Poduslo et al., 1994, St-Amour et al., 2013). Higher expression of CD64 might potentially reflect increased monomeric IgG in the brain, possibly due to systemic infection/inflammation in schizophrenia (Minett et al., 2016, Rakic et al., 2018). Indeed, a previous study has found increased CD64 mRNA transcripts, as well as complement mRNAs, in the midbrain of schizophrenia cases with high level of peripheral inflammation (Purves-Tyson et al., 2020). Interestingly, expression of the activating FcγRs CD32a and CD16 which have low affinity for monomeric IgG and high affinity for immune complexes was unchanged, suggesting that IgG present in the brain is not in the form of immune complexes. Another potential explanation for the increased CD64 expression could be the autoimmune hypothesis of schizophrenia (Lennox et al., 2017). An increased prevalence of autoantibodies targeting peripheral and/or central organs, such as N-methyl-D-aspartate receptor (NMDAR) has been reported in the cortex and blood of individuals with schizophrenia (Al-Diwani et al., 2017, Glass et al., 2017, Pearlman and Najjar, 2014), as well as a bidirectionally increased risk of comorbidity between schizophrenia and autoimmune diseases in general (Benros et al., 2014a, Benros et al., 2014b, Notter, 2018).

None of the other microglial markers show changes in their expression, consistent with previous findings from the same cohorts that at the time of death, microglia do not appear to be involved in aberrant synaptic engulfment in schizophrenia (Tzioras et al., 2021).

The increased CD64 expression was specific to the cerebral cortex. Indeed we observed that, unlike controls, CD64 grey and white matter values did not correlate in schizophrenia patients, supporting previous reports that human grey and white matter microglial populations can differ in their responses (Mittelbronn et al., 2001, Sankowski et al., 2019, van der Poel et al., 2019). Therefore, we hypothesize that enhanced FcγR-dependent activity of microglia is associated with neuronal network disruption. An imbalance in inhibitory (g-aminobutyric acid, GABA) vs. excitatory (NMDA, AMPA) glutamate receptors has been proposed as an underlying mechanism for psychosis in schizophrenia (Egerton et al., 2012). Interestingly, the presence of psychotic symptoms was associated with an increased ratio of CD64/HLA-DR in the cortex, consistent with the hypothesis of immune dysregulation due to a loss of function or impairment in antibody-antigen presentation, which might be exacerbated in the context of a psychotic episode (De Picker et al., 2017). Consequently, our hypothesis of an immune dysregulation of microglia via the Fcγ receptors, encompasses the microglial hypothesis of the aetiology of schizophrenia (Monji et al., 2009) and the presence of abnormal neural oscillations in schizophrenia (Uhlhaas and Singer, 2010), neural disturbances that can be triggered by activated/primed microglia (Ta et al., 2019) and/or a dysregulation in the GABA and NMDA receptor-mediated neural transmission (Uhlhaas and Singer, 2010).

Our current findings point towards an important effect of age on the expression of several microglial markers, consistent with studies showing microglial changes with ageing at the level of the transcriptome, morphology and functions (Cribbs et al., 2012, Davies et al., 2017, Hickman et al., 2013, Olah et al., 2018). A similar positive correlation has been observed between age and TSPO PET binding estimates in healthy subjects and in schizophrenia (Conen et al., 2020, Tuisku et al., 2019). We found an age-dependent increase of Iba1 expression which was only present in patients who died during psychosis, and not in patients without psychosis or controls. Remarkably, this interaction between age- and state-dependent effects appears to mimic our earlier findings of an interaction between age and psychotic state in a longitudinal TSPO PET study in patients with psychotic disorders (De Picker et al., 2019b). While the exact role and dynamic expression of TSPO remains poorly understood, preclinical work has indicated that *in vivo* TSPO expression on PET is most closely represented by Iba1 expression in tissue, and thus the motile function of microglia (Franco-Bocanegra et al., 2019). Our current findings imply the existence of a functional link between TSPO and Iba1 in schizophrenia patients. In younger cross-sectional patient cohorts, the TSPO expression was however found to be unchanged or even decreased (Notter, 2018, Plavén-Sigray et al., 2018, Plavén-Sigray et al., 2020). Decreased TSPO binding was also found in a recent *meta*-analysis on TSPO binding with second generation tracers in psychotic disorders (Plavén-Sigray et al., 2020).

### 4.2 T Lymphocytes and perivascular macrophages in schizophrenia

Fewer schizophrenia cases had CD3 + T lymphocytes in the leptomeninges, but there was no significant difference in the brain parenchyma between the groups. A potential explanation is reduced patrolling of T lymphocytes in the subarachnoid space due to alterations in systemic inflammation/autoimmunity (Gebhardt et al., 2013) associated with schizophrenia (Lennox et al., 2017). However, this finding could have been confounded by the relatively higher age of the controls and therefore needs to be interpreted with care. Infiltration of T lymphocytes has been reported in the hippocampus of patients with residual schizophrenia (Busse et al., 2012) supporting the immune alteration in schizophrenia; however further studies are required to understand the role of the recruited T lymphocytes in the brain. The presence of perivascular macrophages in the parenchyma distant from blood vessels in schizophrenia, previously reported by others (Cai et al., 2018), was not confirmed.

### 4.3 Microglial morphology in schizophrenia

Our detailed assessment of 3D reconstructed human microglia showed larger and less spherical cell bodies in schizophrenia, indicating a more reactive/primed microglial phenotype. Microglial priming following an acute inflammatory event increases their susceptibility to a secondary stimulus, which can trigger a prolonged and exaggerated inflammatory response (Perry and Holmes, 2014). Repetitive inflammatory stimuli move primed microglia to a “trained” phenotype with long-term consequences including cell reprogramming leading to either increased (primed) or decreased (tolerant) immune responses. This process is defined as microglial “immune memory” (Neher and Cunningham, 2019, Wendeln et al., 2018), and is relevant to the human lifespan with microglia reported to live up to 20 years (Reu et al., 2017). This raises the possibility that in schizophrenia, the microglial responses accompanying each subsequent psychotic event increase with age. Indeed, the course of schizophrenia typically changes from a relapsing-remitting phenotype to a more chronic gradual deterioration around 5 years after illness onset (Rosen and Garety, 2005, Wieselgren and Lindstrom, 1996). This hypothesis could also explain some of the observed discrepancies in the TSPO PET signal detected in patients: TSPO binding is unchanged or even decreased early in the illness course, for instance in ultra-high risk of psychosis (Di Biase et al., 2017, Hafizi et al., 2017), first-episode patients (Hafizi et al., 2017) and patients within the first 5 years after illness onset, regardless of clinical state. On the other hand, patients with chronic schizophrenia appear to demonstrate no changes in TSPO uptake outside of acute events, or increased uptake during symptomatic episodes of psychosis (Bloomfield et al., 2016, Conen et al., 2020, De Picker et al., 2019b, Di Biase et al., 2017, Takano et al., 2010) due to primed microglia.

### 4.4 Limitations of the study

Our study has generated new hypotheses on the microglial phenotype in schizophrenia patients, which will need to be confirmed in follow-up studies. Inherent limitations of a retrospective observational study meant it was not possible to explore temporal aspects, with the analysis limited to assessment of late-stage consequences of schizophrenia. Furthermore, as expected, schizophrenia patients died at a relatively younger age and were predominantly of male gender, while the control group also included some individuals of highly advanced age. We controlled for this problem in a sensitivity analysis excluding the oldest control subjects. The *post-mortem* delay was relatively high but similar between the 2 groups. Gender was not included as covariate due to the low number of female schizophrenia cases who had psychotic symptoms at death (n = 3 of 16). Furthermore, we could not determine the potential impact of subjects’ cause of death. In particular death by suicide has been associated with increased HLA-DR expression in the dorsolateral prefrontal cortex, anterior cingulate cortex and thalamus in schizophrenia (Steiner et al., 2008), although a more recent and larger study did not find an increased microglial density in the dorsal raphe nucleus of schizophrenia patients who died by suicide (Brisch et al., 2017). Our data did not allow us to investigate this question due to the limited number of subjects who died by suicide, nor were we able to control for the presence of systemic inflammatory states or eliminate the possibility that our results could be partly affected by exposure to antipsychotic drugs (Notter, 2018). While most patients’ records had some evidence of patients being treated with antipsychotic medication at some point, the records did not allow us to comprehensively gauge lifetime cumulative exposure nor chlorpromazine equivalent dosages of antipsychotics.

## 5 Conclusion

Taking in consideration the limitations mentioned above, our study of immune cells in the human brain shows changes in the expression of the FcγR highlighting the importance of communication between the central and systemic immune system in schizophrenia. Patients in whom psychotic symptoms were present at time of death demonstrated age-dependent increases of Iba1 and increased CD64/HLA-DR ratios. We were also able to observe a potential role for T lymphocytes, but we did not confirm the presence of recruited macrophages in the brains of schizophrenia patients. Microglial morphology tended towards a primed/reactive morphology, consistent with the hypothesis of an alteration of the immune environment. Overall, brain inflammation in chronic schizophrenia appears to low-grade, with state- and age-dependent effects.

## Funding

This work was supported by the the Medical Research Council (UKRI, G1100578) and Alzheimer’s Research UK (grant number ARUK-EG2015A-4); the Mexican National Council of Science and Technology (CONACyT reference 899569) to GMV and the Flemish Agency for Innovation by Science and Technology (IWT-SB reference 121373) to LDP.

## Declaration of Competing Interest

The authors declare that they have no known competing financial interests or personal relationships that could have appeared to influence the work reported in this paper.
