# Supplementary material for: Immune environment of the brain in schizophrenia and during the psychotic episode: A human post-mortem study
Source: Brain Behav Immun. 2021 Oct;97:319–27. doi: 10.1016/j.bbi.2021.07.017 (PMC8475749; doi:10.1016/j.bbi.2021.07.017)
Supplement: Supplementary data 1 [file mmc1.docx]

**Supplementary information**

**Supplementary Table 1:** Characteristics of the primary antibodies, immunohistochemistry conditions and expression of the staining

| **Antigen** | **Company** | **Host** | **Pre-treatment** | **Dilution** | **Predominant neuropathological features / cell type stained** | **Immune functions** |
| --- | --- | --- | --- | --- | --- | --- |
| HLA-DP, DQ, DR | Dako | Mouse (clone CR3/43) | Citrate buffer | 1/200 | Microglia, perivascular macrophages | HLA-DR is a Major Histocompatibility Class (MHC) II cell surface receptor which presents antigens to cells of the immune system eliciting an immune response, involved in the non-self-recognition and upregulated in inflammation (Styren et al., 1990). |
| Iba1 | Wako | Rabbit | Citrate buffer | 1/1000 | Microglia, perivascular macrophages | Cytoplasmic protein involved in cytoskeletal reorganization, membrane ruffling of the microglial processes and actin cross-linking needed for cell migration (Ohsawa et al., 2004), reflecting microglial motility and migration properties (Franco-Bocanegra et al., 2019b) |
| CD68 | Dako | Mouse (clone  PG-M1) | Citrate buffer | 1/500 | Microglia, perivascular macrophages | CD68 labels lysosomal and endosomal transmembrane glycoprotein of microglia, indicating phagocytic activity (Rabinowitz and Gordon, 1991). |
| P2RY12 | Sigma Aldrich | Rabbit | Citrate buffer | 1/200 | Microglia, perivascular macrophages | P2RY12 is a purinergic receptor (Franco-Bocanegra et al., 2019a) identified as part of the physiological/homeostatic signature of microglia (Butovsky et al., 2014). |
| CD64 (FcγRI) | R&D Systems | Goat | EDTA buffer | 1/100 | Microglia, perivascular macrophages | Fcγ receptors are central effectors of immunoglobulin (IgG) mediated immune responses (Nimmerjahn et al., 2015). CD64 has high affinity for the Fc portion of IgG, triggering a monocyte/macrophage response (Vogelpoel et al., 2015). CD64 expression reflects the presence of IgG in the brain and thus the involvement of systemic immunity (Lunnon et al., 2011). |
| CD32a (FcγRIIa) | Abcam | Mouse (clone 13D7) | EDTA buffer | 1/2000 | Microglia, perivascular macrophages and in some neurons | CD32a is a low/medium affinity activating receptor for immune complex (Nimmerjahn et al., 2015; Nimmerjahn and Ravetch, 2008). |
| CD32b (FcγRIIb) | Abcam | Rabbit (clone EP888Y) | EDTA buffer | 1/4000 | Neurons | CD32b is the only inhibitory receptor with a low/medium affinity inhibitory receptor for immune complex (Hogarth and Pietersz, 2012; Ono et al., 1996). |
| CD16 (FcγRIII) | R&D Systems | Goat | EDTA buffer | 1/150 | Microglia, perivascular macrophages, some monocytes and neurons | CD16 is a low/medium affinity activating receptor for immune complex (Nimmerjahn et al., 2015). |
| CD163 | Serotec | Mouse (clone EDHu-1) | EDTA buffer | 1/1000 | Perivascular macrophages, sparse microglia and monocytes | CD163 is a membrane glycoprotein belonging to the scavenger receptor cysteine-rich (SRCR) superfamily group B, which functions as a high-affinity receptor for the haemoglobin-haptoglobin complex (Fabriek et al., 2007). |
| CD206 | Abcam | Mouse (clone 5C11) | EDTA buffer | 1/4000 | Perivascular macrophages | CD206 is a mannose receptor that is involved in pathogen recognition and receptor-mediated endocytosis (Linehan et al., 2000). |
| CD3 | Dako | Mouse (clone F7.2.38) | None | 1/200 | T lymphocytes | CD3 is a T-cell co-receptor necessary to activate both CD8^+^ and CD4^+^ T cells (Chetty and Gatter, 1994), and thus considered as a pan-T cell marker. |

**Supplementary Table 2:** Correlations between the different microglial markers in control and schizophrenia cases

|  | **Grey matter** | | | **White matter** | |  |
| --- | --- | --- | --- | --- | --- | --- |
|  | **Ctrl** | **Sz** | **Ctrl** | | **Sz** | |
| HLA-DR *vs* CD68 | ρ=-0.223, p=0.185 | ρ=-0.060, p=0.722 | ρ=-0.123, p=0.467 | | ρ=0.020, p=0.905 | |
| HLA-DR *vs* Iba1 | ρ=0.375, p=0.020 | ρ=0.364, p=0.032 | ρ=0.315, p=0.054 | | ρ=0.334, p=0.050 | |
| HLA-DR *vs* CD64 | ρ=0.174, p=0.341 | ρ=0.344, p=0.058 | ρ=0.080, p=0.663 | | ρ=0.358, p=0.052 | |
| HLA-DR *vs* CD32a | ρ=0.205, p=0.231 | ρ=0.128, p=0.471 | ρ=0.392, p=0.020 | | ρ=-0.160, p=0.372 | |
| HLA-DR *vs* CD32b | **ρ=0.571, p<0.001** | ρ=0.438, p=0.012 | **ρ=0.448, p=0.009** | | ρ=0.414, p=0.018 | |
| HLA-DR *vs* CD16 | **ρ=0.496, p=0.003** | **ρ=0.546, p=0.001** | **ρ=0.533, p=0.001** | | ρ=0.331, p=0.052 | |
| HLA-DR *vs* P2RY12 | **ρ=0.576, p<0.001** | **ρ=0.552, p<0.001** | **ρ=0.666, p<0.001** | | **ρ=0.508, p=0.002** | |
| CD68 *vs* Iba1 | ***r=-0.460, p=0.004*** | ρ=0.413, p=0.014 | *r=-0.243, p=0.148* | | *r=-0.195, p=0.262* | |
| CD68 *vs* CD64 | ρ=0.072, p=0.699 | ρ=0.354, p=0.051 | *r=-0.344, p=0.058* | | ρ=0.098, p=0.608 | |
| CD68 *vs* CD32a | ρ=-0.133, p=0.445 | ρ=-0.005, p=0.977 | ρ=-0.114, p=0.514 | | ρ=0.243, p=0.172 | |
| CD68 *vs* CD32b | ρ**=-0.480, p=0.005** | ρ=0.367, p=0.039 | ρ=-0.225, p=0.216 | | ρ=-0.007, p=0.968 | |
| CD68 *vs* CD16 | ρ=-0.062, p=0.730 | ρ=-0.028, p=0.872 | ρ=0.037, p=0.840 | | ρ=-0.142, p=0.415 | |
| CD68 *vs* P2RY12 | ρ=-0.302, p=0.065 | ρ=-0.244, p=0.151 | ρ=-0.315, p=0.054 | | ρ=-0.083, p=0.629 | |
| Iba1 *vs* CD64 | ρ=0.187, p=0.313 | ρ=0.022, p=0.910 | *r=-0.103, p=0.581* | | ρ=0.153, p=0.429 | |
| Iba1 *vs* CD32a | ρ=0.387, p=0.020 | ρ=0.004, p=0.981 | ρ=0.359, p=0.034 | | ρ=0.078, p=0.675 | |
| Iba1 *vs* CD32b | **ρ=0.704, p<0.001** | **ρ=0.487, p=0.006** | **ρ=0.696, p<0.001** | | ρ=0.377, p=0.040 | |
| Iba1 *vs* CD16 | ρ=0.139, p=0.433 | ρ=0.369, p=0.034 | ρ=0.356, p=0.039 | | **ρ=0.499, p=0.003** | |
| Iba1 *vs* P2RY12 | **ρ=0.579, p=0<0.001** | ρ=0.297, p=0.088 | **ρ=0.633, p<0.001** | | **ρ=0.454, p=0.007** | |
| CD64 *vs* CD32a | ρ=-0.113, p=0.553 | ρ=0.103, p=0.595 | ρ=-0.067, p=0.725 | | ρ=-0.027, p=0.893 | |
| CD64 *vs* CD32b | ρ=-0.109, p=0.579 | ρ=-0.045, p=0.828 | ρ=-0.242, p=0.223 | | ρ=0.410, p=0.037 | |
| CD64 *vs* CD16 | ρ=0.005, p=0.979 | ρ=0.241, p=0.192 | ρ=0.070, p=0.725 | | ρ=0.336, p=0.070 | |
| CD64 *vs* P2RY12 | ρ=0.145, p=0.429 | ρ=0.338, p=0.063 | ρ=0.050, p=0.786 | | ρ=0.220, p=0.242 | |
| CD32a *vs* CD32b | ρ=0.287, p=0.111 | ρ=0.116, p=0.551 | ρ=0.126, p=0.508 | | ρ=0.137, p=0.488 | |
| CD32a *vs* CD16 | ρ=0.341, p=0.056 | **ρ=0.495, p=0.004** | ρ=0.177, p=0.333 | | ρ=0.285, p=0.121 | |
| CD32a *vs* P2RY12 | ρ=0.054, p=0.752 | ρ=-0.021, p=0.906 | ρ=0.166, p=0.335 | | ρ=0.152, p=0.406 | |
| CD32b vs CD16 | ρ=0.347, p=0.065 | **ρ=0.485, p=0.007** | ρ=0.337, p=0.080 | | **ρ=0.475, p=0.007** | |
| CD32b *vs* P2RY12 | **ρ=0.705, p<0.001** | **ρ=0.650, p<0.001** | **ρ=0.612, p<0.001** | | **ρ=0.554, p=0.001** | |
| CD16 *vs* P2RY12 | ρ=0.340, p=0.049 | ρ=0.184, p=0.291 | ρ=0.407, p=0.017 | | ρ=0.246, p=0.153 | |

Bold: significant p-value ≤0.01; Italic: Pearson’ *r* and p-value; Non-italic: Spearman’s ρ and p-value. *Ctrl*, neurologically/cognitively normal controls; *Sz*, Schizophrenia cases; ns, non-significant.

**References**

Chetty, R., Gatter, K., 1994. CD3: structure, function, and role of immunostaining in clinical practice. J Pathol 173, 303-307.

Fabriek, B.O., Polfliet, M.M., Vloet, R.P., van der Schors, R.C., Ligtenberg, A.J., Weaver, L.K., Geest, C., Matsuno, K., Moestrup, S.K., Dijkstra, C.D., van den Berg, T.K., 2007. The macrophage CD163 surface glycoprotein is an erythroblast adhesion receptor. Blood 109, 5223-5229.

Franco-Bocanegra, D.K., George, B., Lau, L.C., Holmes, C., Nicoll, J.A.R., Boche, D., 2019a. Microglial motility in Alzheimer's disease and after Abeta42 immunotherapy: a human post-mortem study. Acta Neuropathol Commun 7, 174.

Hogarth, P.M., Pietersz, G.A., 2012. Fc receptor-targeted therapies for the treatment of inflammation, cancer and beyond. Nat Rev Drug Discov 11, 311-331.

Linehan, S.A., Martinez-Pomares, L., Gordon, S., 2000. Mannose receptor and scavenger receptor: two macrophage pattern recognition receptors with diverse functions in tissue homeostasis and host defense. Adv Exp Med Biol 479, 1-14.

Lunnon, K., Teeling, J.L., Tutt, A.L., Cragg, M.S., Glennie, M.J., Perry, V.H., 2011. Systemic inflammation modulates Fc receptor expression on microglia during chronic neurodegeneration. J Immunol 186, 7215-7224.

Nimmerjahn, F., Ravetch, J.V., 2008. Fcgamma receptors as regulators of immune responses. Nat Rev Immunol 8, 34-47.

Ohsawa, K., Imai, Y., Sasaki, Y., Kohsaka, S., 2004. Microglia/macrophage-specific protein Iba1 binds to fimbrin and enhances its actin-bundling activity. J Neurochem 88, 844-856.

Ono, M., Bolland, S., Tempst, P., Ravetch, J.V., 1996. Role of the inositol phosphatase SHIP in negative regulation of the immune system by the receptor Fc(gamma)RIIB. Nature 383, 263-266.

Rabinowitz, S.S., Gordon, S., 1991. Macrosialin, a macrophage-restricted membrane sialoprotein differentially glycosylated in response to inflammatory stimuli. J Exp Med 174, 827-836.

Styren, S.D., Civin, W.H., Rogers, J., 1990. Molecular, cellular, and pathologic characterization of HLA-DR immunoreactivity in normal elderly and Alzheimer's disease brain. Exp Neurol 110, 93-104.

Vogelpoel, L.T., Baeten, D.L., de Jong, E.C., den Dunnen, J., 2015. Control of cytokine production by human fc gamma receptors: implications for pathogen defense and autoimmunity. Front Immunol 6, 79.

**Supplementary Table** **3:** Correlations of the immunomarkers between the grey and white matter.

| **grey *vs* white matter** | **HLA-DR** | **Iba1** | **CD68** | **P2RY12** | **CD64** | **CD32a** | **CD16** |
| --- | --- | --- | --- | --- | --- | --- | --- |
| **Ctrl** | **ρ=0.946**  **P<0.001** | **r=0.874**  **P<0.001** | **r=0.798**  **P<0.001** | **ρ=0.929**  **P<0.001** | **ρ=0.748**  **P<0.001** | **ρ=0.574**  **P<0.001** | **ρ=0.821**  **P<0.001** |
| **Sz** | **ρ=0.911**  **P<0.001** | **ρ=0.908**  **P<0.001** | **r=0.790**  **P<0.001** | **ρ=0.924**  **P<0.001** | ρ=0.371  P=0.044 | ρ=0.297  P=0.093 | **ρ=0.796**  **P<0.001** |

ρ, Spearman; r, Pearson; bold: significant p-value ≤0.01

*Ctrl*, neurologically/cognitively normal controls; *Sz*, Schizophrenia cases

**Supplementary Table 4.** CD3+ T lymphocyte quantification in schizophrenia and control cases

|  | **Ctrl (n=40)** | **Sz (n=37)** | **P value** |
| --- | --- | --- | --- |
| **Perivascular compartment** |  |  |  |
| Grey matter | 29/39 (74.4%) | 25/35 (71.4%) | 0.799 |
| White matter | 27/39 (69.2%) | 26/36 (72.2%) | 0.805 |
| **Parenchyma** |  |  |  |
| Grey matter | 18/39 (46.2%) | 25/36 (69.4%) | 0.061 |
| White matter | 15/39 (38.5%) | 19/36 (52.8%) | 0.251 |
| **Meninges** | 11/39 (28.2%) | 3/36 (8.3%) | *0.038* |
|  | 5/31 (16,1%) | 3/36 (8.3%) | 0.456 |

Fisher t-test, Significant p value in italic

*Ctrl*, neurologically/cognitively normal controls; *Sz*, Schizophrenia cases.

Data presented as number (percentage %) of cases with CD3+ T cells in the investigated compartment
